# Supplementary material for: COVID-19 knowledge, attitudes, and practices of United Arab Emirates medical and health sciences students: A cross sectional study
Source: PLoS One. 2021 May 12;16(5):e0246226. doi: 10.1371/journal.pone.0246226 (PMC8115851; doi:10.1371/journal.pone.0246226)
Supplement: S1 Appendix — (PDF) [file pone.0246226.s001.pdf]

A Cross Sectional Study among Medical and Health Sciences Students in the United Arab Emirates: Knowledge, Attitudes, and Practices of COVID-19

Online Questionnaire

Part 1: Sociodemographic data

1.1 Age (in years):

[ ]

1.2 Gender:

- Male
- Female

1.3 Nationality: (if you choose other, please specify)

- UAE citizen
- Other, please specify

[ ]

1.4 Marital Status:

- Single
- Married
- Divorced
- Widowed

1.5 Do you have any children?

- Yes
- No

1.6 Emirate of residence:

- Abu Dhabi
- Dubai
- Ajman
- Sharjah

- Fujairah
- Ras Al Khaimah
- Umm Al Quwain

1.7 How many people live in your household including housemaids?

1.8 Which University do you attend?

- UAE University
- Khalifa University
- Mohammed Bin Rashid University
- Dubai Medical College
- University of Sharjah
- Gulf Medical University
- Ajman University
- RAK Medical and Health Sciences University
- Abu Dhabi University
- Fatima College of Health Sciences
- New York University Abu Dhabi
- Higher Colleges of Technology
- Other, please specify

1.9 What is your current field of specialty?

- Medicine
- Nursing
- Health emergency (Paramedics)
- Pharmacy
- Radiography and medical imaging
- Physiotherapy
- Medical Laboratory Technology
- Dental
- Public health
- Biomedical sciences
- Other, please specify

1.10 Type of degree (study program):

- o Undergraduate
- o Postgraduate

1.11 Total duration of the study program you are enrolled in (in years):

[ ]

1.12 Year of study:

- o Year 1
- o Year 2
- o Year 3
- o Year 4
- o Year 5
- o Year 6

1.13 Do you suffer from any chronic diseases?

- o Yes
- o No

1.14 If you answer to the previous question was yes, please specify:

- o Diabetes
- o Hypertension
- o Asthma
- o Other, please specify

[ ]

1.15 Do you live with someone who has a chronic disease (such as Respiratory diseases/Asthma, Diabetes, Cardiovascular disease, renal disease, liver diseases etc.)?

- o Yes
- o No

1.16 If your answer to the previous question was yes, please specify:

[ ]

1.17 Did any of your family members or friends were tested for COVID-19?

- Yes
- If yes, was it?
    - positive
    - Negative
- No

1.18 If your answer the previous question was yes, what was the outcome?

- Asymptomatic
- Mild symptoms, isolated
- Severe symptoms and admitted to the hospital
- Severe symptoms and admitted in the Intensive Care Unit (ICU)
- Died
- Other, please specify

1.19 Have you been tested for COVID-19?

- Yes
- No

1.20 If your answer to the previous question was yes, please specify the result:

- Positive
- Indeterminate
- Negative
- Not known

1.21 What is your source of COVID-19 information? (*you can select more than one option*)

- TV
- Social Media
- Healthcare professionals
- Colleagues
- Your university
- Online courses (such as Webinars)
- Medical databases/websites
- Other, please specify

1.22 Have you attended any COVID-19 education courses (webinars/seminars)?

- Yes
- No

<u>Part 2: Knowledge, Attitude and Practice</u>

Section 1: Knowledge about COVID-19

2.1 COVID-19 is a new disease caused by virus SARS-CoV-2:

- True
- False
- Don't know

2.2 Which animal is most likely to transmit this virus to human? (*you can select more than one option*)

- Bat
- Camel
- Pangolin
- Civet cat
- House pets
- Don't know

2.3 SARS-CoV-2 can be transmitted between humans by the following routes? (*you can select more than one option*):

- Respiratory droplet
- Airborne
- Surfaces
- Vector (such as mosquitoes, flies, lice, etc)
- Bodily fluids (such as blood, saliva, etc)

2.4 Which of the following are COVID-19 symptoms? (*you can select more than one option*)

- Fever
- Dry cough
- Cough with sputum

- Shortness of breath
- Nausea, vomiting, diarrhea
- Seizure
- Loss of taste
- Runny nose
- Loss of sense of smell
- Don’t know

2.5 What is the average incubation period of COVID-19? *(select only one option)*

- 1-3 days
- 4-5 days
- 5-7 days
- 7-14 days
- Don’t know

2.6 What is the best diagnostic test for COVID-19? *(select only one option)*

- RT-PCR (using nasopharyngeal swab or oropharyngeal swab)
- Serology (Antibody test)
- Blood culture
- Lung CT scan
- Don’t know

2.7 COVID-19 can be treated by using the following (*you can select more than one option*):

- Anti-viral
- Anti-malarial
- Antibiotics
- Convalescent plasma transfusion
- Don’t know

2.8 Which of the following can reduce the spread of COVID-19? (*you can select more than one option*)

- Social distancing
- Self-isolation
- Wearing face masks
- Herbal remedies (ex: ginger, thyme, chamomile, turmeric, etc)

- Avoiding crowded places
- Don’t know

2.9 People who are asymptomatic and COVID-19 test positive must stay at home until they are free of the infection:

- True
- False
- Don’t know

2.10 Generally, who should wear N95 masks? *(select only one option)*

- Everyone
- Healthcare professionals dealing with COVID-19 patients
- Someone who is tested positive for COVID-19
- Don’t know

2.11 Persons with COVID-19 cannot transmit the virus to others when a fever is not present:

- True
- False
- Don’t know

2.12 It is not necessary for children and young adults to take measures to prevent infection from COVID-19 virus:

- True
- False
- Don’t know

Section 2: Attitude

3.1 You are worried that you will get COVID-19 *(select only one option)*:

- Strongly agree
- Agree
- Disagree
- Strongly disagree
- No opinion

3.2 You are worried that a family member can get infected with this virus *(select only one option)*:

- Strongly agree
- Agree
- Disagree
- Strongly disagree
- No opinion

3.3 Infection with the virus is associated with stigma (for example: the infected people feel ashamed because people are afraid of them and therefore avoid them) *(select only one option)*:

- Strongly agree
- Agree
- Disagree
- Strongly disagree
- No opinion

3.4 The current measures taken by the UAE government are effective in stopping the spread of the infection *(select only one option)*:

- Strongly agree
- Agree
- Disagree
- Strongly disagree
- No opinion

3.5 In your opinion, is there anything that can be done more?

3.6 You are confident that the UAE will be able to stop the spread of the virus *(select only one option)*:

- Strongly agree
- Agree
- Disagree
- Strongly disagree
- No opinion

Section 3: Practice

4.1 In the last 2 weeks, have you visited any of the following places? (*you can select more than one option*)

- o Shopping malls
- o Grocery shops or supermarkets
- o Barber shops or beauty salons
- o Friends or family gatherings
- o Restaurants or coffee shops
- o Industrial areas
- o Hospitals for treatment purposes
- o COVID-19 facilities for volunteering purposes (hospitals/testing centers/hotels/etc.)
- o I have not left my home

4.2 What precautions do you take when accepting home deliveries? (*you can select more than one option*)

- o None
- o Wearing masks
- o Wearing gloves
- o Disinfecting packages
- o Hand washing after accepting packages
- o Storing items in a room for few days before using them
- o Other, please specify

4.3 Since the pandemic started, have you been washing your hands more frequently?

- o Yes
- o No

4.4 If you answer to the previous question was yes, please choose in which situation (*you can select more than one option*):

- o After using the toilet
- o Before cooking
- o Before eating
- o When coming from outside

- After touching more frequently on surfaces
- Other, please specify

4.5 Since the pandemic started, have you been wearing face masks?

- Yes
- No

4.6 If your answer to the previous question was yes, in which situations do you wear a face mask? (*you can select more than one option*)

- In the car
- In the shopping mall
- When walking down the street
- When going to the grocery shop
- When going to the mosque
- When visiting friends or family members
- When going to the hospital
- When accepting home deliveries
- Other, please specify

4.7 Do you follow the curfew timings set by the UAE government?

- Yes
- No

4.8 With whom did you discuss COVID-19 since the pandemic started? (*you can select more than 1 option*)

- Family members
- Friends
- Colleagues at school/college
- Faculty members
- Social media platforms (such as twitter, snapchat, Instagram, etc)
- Other
