# Supplementary material for: COVID-19 knowledge, attitudes, and practices of United Arab Emirates medical and health sciences students: A cross sectional study
Source: PLoS One. 2021 May 12;16(5):e0246226. doi: 10.1371/journal.pone.0246226 (PMC8115851; doi:10.1371/journal.pone.0246226)
Supplement: Attachment — Submitted filename: Response to reviewers.docx [file pone.0246226.s003.docx]

4^th^ April 2021

Professor Jenny Wilkinson

Academic Editor

PLOS ONE

***Ref PONE-D-21-01418: COVID-19 Knowledge, Attitudes, and Practices of United Arab Emirates Medical and Health Sciences Students: A Cross Sectional Study***

Dear Professor Wilkinson

Thank you for your prompt response and giving us the chance to re-submit a revised version of the above manuscript. The manuscript has now been completely re-written as advised by the reviewers. We thank you and the reviewers for their highly encouraging and constructive comments, which have significantly improved our manuscript. All changes made in the manuscript are highlighted in yellow color to facilitate the review process. The answers to the Editorial and reviewers’ comment are as follows:

**Journal Requirements**

**Comment 1.** Please ensure that your manuscript meets PLOS ONE's style requirements, including those for file naming.

**Answer:** We confirm that our manuscript meets PLOS ONE's style requirements.

**Comment 2.** We note that you have stated that you will provide repository information for your data at acceptance. Should your manuscript be accepted for publication, we will hold it until you provide the relevant accession numbers or DOIs necessary to access your data.

**Answer:** The information is submitted as an Excel supplementary file which can be published when the paper is accepted.

**Comment 3.** Your ethics statement should only appear in the Methods section of your manuscript. If your ethics statement is written in any section besides the Methods, please delete it from any other section.

**Answer:** We confirm that the ethics statement is written only in the Methods section.

**Comment 4.** We note you have included a table to which you do not refer in the text of your manuscript. Please ensure that you refer to Table 5 in your text; if accepted, production will need this reference to link the reader to the Table.

**Answer:** Table 5 (now Table 6) has now been cited in the manuscript (Page 16).

**Comment 5.** Please include additional information regarding the survey or questionnaire used in the study and ensure that you have provided sufficient details that others could replicate the analyses. For instance, if you developed a questionnaire as part of this study and it is not under a copyright more restrictive than CC-BY, please include a copy, in both the original language and English, as Supporting Information.

**Answer**: The survey is now attached as a supplementary file (**Appendix 1**, Page 6, Paragraph 3) and more details about its development are added as requested (Page 6, Paragraph 2).

**Comment 6**: Furthermore, please provide additional information regarding the validation of the questionnaire.

**Answer**: This has now been answered in comment 3 of the Editor and discussed in the limitations of the study (Page 6, Paragraph 2; Page 21, last paragraph).

**Additional Editor Comments**

Thank you for your submission, two reviewers’ comments are attached. As one of the reviewers provided very brief comments, I have provided additional comments.

**Comment 1.** While the language in the work is generally acceptable it would benefit from careful proof reading to ensure correct grammar and sentence structure is used

**Answer:** Thank you for your advice. The manuscript has now been edited by a professional English language Lecturer. Kindly note the acknowledgment section (Page 24) and attached certificate of linguistic editing.

**Comment 2.** There is a large and growing body of literature on COVID impacts on students and their attitudes etc, this is not reflected in the Introduction to your work and the work would be strengthen by a more comprehensive review of literature on this topic.

Answer: Thank you for your kind advice. The introduction has now been modified as suggested by performing a comprehensive review of the literature (Pages 3 and 4).

**Comment 3.** Please provide more detailed information about the construction and testing of the survey instrument, for example what is the validity and reliability of the instrument?

**Answer:** Thank you for your important comment. We have now provided more information about the construction and testing of the survey instrument as requested and added it as an appendix (Page 6, Paragraph 2; appendix 1).

**Comment 4.** The statistical analysis is rather superficial as it is presented as basis descriptive statistics – the work would be strengthened by more comprehensive analysis.

**Answer:** Thank you for your advice which is highly appreciated. We have now quantified the knowledge and practice (methods, Page 7, Paragraph 3) and compared the results of knowledge (Page 12, last paragraph), attitudes (Page 15) and practices (Page 18) between medical students and allied health students and between undergraduate with postgraduate students as requested. This resulted in an extra table (Table 5, Pages 15 and 16); and two new figures (Figures 1, Page 13; and Figure 2, Page 18).

**Comment 5**. In Table 1 a number of institutions are listed however it is unclear what type of sampling method was used to ensure that the sample was representative

**Answer:** Thank you for your comment. As we did not have an access to the information about the number of students at relevant institutions we were not able to estimate a sample size. As the method of data collection was an online-based survey, we used a non-probability sampling approach, namely convenience sampling. This has now been clarified (Page 5, Paragraph 3).

**Comment 6.** The column titles are incorrect for the age item in Table 1

**Answer:** The table has been corrected as advised. All variables are expressed as number (%) except age which is expressed as median (range); (Page 8, Page 9 footnote)

**Comment 7.** In Table 3 where questions have multiple answers these should be provided so readers know what is selected as correct or incorrect

**Answer:** The correct answers have been incorporated in the Table 3 as recommended (Pages 11 and 12).

**Comment 8.** With strengthening and improvements to the Introduction and analysis this will allow the Discussion to be recrafted to provide a more comprehensive and critical discussion of the findings

**Answer:** The discussion has been modified as advised to be more comprehensive and critical to our findings (Page 19-21).

**Reviewers' comments**

**Reviewer 1**

**Comment:** The research topic and methodology is similar to research entitled: A Cross-Sectional Study on University Students’ Knowledge, Attitudes, and Practices Toward COVID-19 in the United Arab Emirates, Am. J. Trop. Med. Hyg., 104(1), 2021, pp. 75–84, doi:10.4269/ajtmh.20-0857. I think this research don have primary conditions for publishing.

**Answer:** Thank you for your very useful comment which is highly appreciated. We have now critically read the mentioned paper (reference 28 in our paper). It covers only Sharjah University students and aimed to compare COVID19–related KAP between health-related (HR) and non-HR (NHR) majors. It was done during the first two weeks of May 2020 (early of the pandemic) and published end of November 2020 while ours targeted all UAE Medical and Health Sciences students and was run between 2^nd^ June and 19^th^ August 2020 (2 months). It is very common that researchers think of the same important question at the same time. We regret that were not aware of this study once we submitted our paper. We have now included it in the introduction (Page 4, last Paragraph) and discussion (Page 19, Second Paragraph).

**Reviewer 2**

Please consider the following issues:

**Comment 1:** In the keywords section, you should use MeSH terms. KAP is not a suitable term. It is not only an abbreviation, but also a non-MeSH term. Additionally, UAE should be changed. Check out MeSH database to use the correct form of the term.

**Answer:** Thank you for your advice. The key words have been chosen to be MeSH terms (Page 2).

**Comment 2:** The running title is not suitable and it should be revised.

**Answer:** Thank you for your advice. The running title has been changed as advised (Page 1).

**Comment 3:** Introduction is not well-developed. The rationale and necessities of conducting this research is not enough described. Also, more articles are needed in this section.

**Answer:** Thank you for your kind advice. The introduction has now been modified as suggested by performing a comprehensive review of the literature (Pages 3 and 4).

**Comment 4:** “The estimated population of UAE is 9,948,495”. Why did you state this information?! It is not necessary! You can state the reasons. The sample size section should be revised comprehensively.

**Answer:** Thank you for important concern. We have now revised the sample size section as advised (Page 5, last Paragraph). It reads now as follows: “We developed a sampling frame including the list of all medical and health sciences colleges and universities in the UAE. As we did not have an access to the information about the number of students at relevant institutions we were not able to estimate a sample size. As the method of data collection was an online-based survey, we used a non-probability sampling approach, namely convenience sampling”.

**Comment 5:** Information about validity and reliability of the tool should be offered.

**Answer:** We have now provided more information about the validity and reliability of the study tool (Page 6, Paragraph 2) and discussed it in the limitations of the study (Page 21, last Paragraph).

**Comment 6:** Statistical analysis section should be stated in more details.

**Answer:** This section has been detailed as requested (Page 7 paragraphs 3 and 4; and Page 8 Paragraph 1).

**Comment 7:** State response rate in the results.

**Answer:** Thank you for your concern. As the method of data collection was an online-based survey, we used a non-probability sampling approach, namely convenience sampling. Accordingly, we could not know the response rate. We have now highlighted this in the methods (Page 5, last paragraph) and discussed it in the limitations sections (Page 21, Paragraph 2).

**Comment 8:** The style of writing in some parts is not appropriate and not written academically and professionally. Please read and check out the whole article for these issues (e.g. line 136 page 8). Mind grammatical, and dictation issues too.

**Answer:** Thank you for your advice. The manuscript has now been edited by a professional English language Lecturer. Kindly note the acknowledgment section (Page 24) and attached certificate of linguistic editing.

**Comment 9:** Some parts of the discussion are similar to results or suit there. This part should be more narrative and contain more comparative information based on more similar studies. It is not common to state numerical data in this part.

**Answer:** Thank you for you kind advice. We have revised the discussion taking into consideration these points (Pages 19-21).

Thank you for your consideration for this revised manuscript. We hope that these changes will satisfy the reviewers and that the manuscript finally finds a place in your reputable journal.

Yours Sincerely

Mohamud Sheek-Hussein
