# Supplementary material for: COVID-19 knowledge, attitudes, and practices of United Arab Emirates medical and health sciences students: A cross sectional study
Source: PLoS One. 2021 May 12;16(5):e0246226. doi: 10.1371/journal.pone.0246226 (PMC8115851; doi:10.1371/journal.pone.0246226)
Supplement: Attachment — Submitted filename: Response to reviewers.docx [file pone.0246226.s004.docx]

23^rd^ April 2021

Professor Jenny Wilkinson

Academic Editor

PLOS ONE

***Ref PONE-D-21-01418R1: COVID-19 Knowledge, Attitudes, and Practices of United Arab Emirates Medical and Health Sciences Students: A Cross Sectional Study (Second revision)***

Dear Professor Wilkinson

Thank you for your prompt response and giving us the chance to re-submit a second revised version of the above manuscript. The manuscript has now been revised as advised. We thank you and the reviewers for their highly encouraging and constructive comments, which have significantly improved our manuscript. All changes made in the manuscript are highlighted in yellow color to facilitate the review process. The answers to the Editorial and reviewers’ comment are as follows:

**Journal Requirements**

**Comment 1.** Please review your reference list to ensure that it is complete and correct. If you have cited papers that have been retracted, please include the rationale for doing so in the manuscript text, or remove these references and replace them with relevant current references. Any changes to the reference list should be mentioned in the rebuttal letter that accompanies your revised manuscript. If you need to cite a retracted article, indicate the article’s retracted status in the References list and also include a citation and full reference for the retraction notice.

**Answer:** Thank you for your valuable comment. The references have now been changed following exactly your instructions. All changes made in the references are highlighted by yellow. We replaced reference 13 by another recent reference giving the same message which is easier to find in PUBMED. We have also added four needed references to the limitation section (References 40-43).

**Editor’s Comments**

Thank you for your responses to reviewer comments and modifications to the manuscript.

**Comment 1.** On lines 156-158 rather than using the term ‘bad practices’ it is suggested that ‘poor practices’ is used

**Answer:** Thank you for your kind advice. The term has now been corrected as requested (Page 8, line 168).

**Comment 2.** In figure 1 there is a statistical difference shown in A however the practical difference appears small; please comment on whether this is a practical difference. Please also double check that this is in fact a statistically significant difference.

**Answer:**

Thank you for your kind concern. Please be assured that the statistical analysis is correct, and the reporting is accurate. The senior author of the current paper (FA-Z) is the Statistical Editor of World Journal of Emergency Surgery (IF=4.1) and he did the analysis and drew the graphs himself. Kindly note that the overall score was skewed as shown in the figure below and that is why we used nonparametric methods and presented the figures as Box plot and IQ range which is the proper way. Kindly note that the medians are different. The Mann Whitney test compares the ranks, and the difference of the ranks is more than 22% which we consider as a practical difference. This has now been added to the results section as requested (Page 13, line 213).

The results of the SPSS program are shown below.

**Mann-Whitney Test**

| **Ranks** | | | | | | |
| --- | --- | --- | --- | --- | --- | --- |
|  | Specialty | | N | Mean Rank | | Sum of Ranks |
| Knowledge Overall Score | Medicine | | 398 | 394.15 | | 156872.50 |
|  | Other | | 313 | 307.49 | | 96243.50 |
|  | Total | | 711 |  | |  |
|  | | **Knowledge Overall Score** | | |  |  |
| Mann-Whitney U | | 47102.500 | | |  |  |
| Wilcoxon W | | 96243.500 | | |  |  |
| Z | | -5.595 | | |  |  |
| Asymp. Sig. (2-tailed) | | .000 | | |  |  |
| a. Grouping Variable: Specialty | | | | |  |  |

EXAMINE VARIABLES=KnowledgeOverall BY Typeofdegreestudyprogram

**Comment 3.** Please also consider the comments of Reviewer 2 and determine what other changes could be made to improve the work.

**Answer:** Thank you for your important advice which we have genuinely followed. We have tried our best to accommodate as much as possible from the reviewer’s comments within the manuscript. It is obvious that the reviewer recommended rejection depending on the below addressed concerns.

1. The reviewer thinks that the introduction does not lead to a clear aim. We completely disagree with this comment. The introduction has a classical reversed pyramid approach in which we started broad and then narrowed the discussion gradually to reach a well-defined aim at the end of the introduction.
2. The reviewers thinks that this study is similar the study of reference 28 which is not the case at all. Reference 28 studied a single university in UAE with both medical and non-medical students while we have studied medical and allied health students in all UAE. Our study has much more generalizability compared with reference 28. We have clarified this in the introduction (Page 4, lines 91-93).
3. The reviewer based his rejection on the basis that the methods are not novel. We have submitted our paper to PLOS ONE because it does not consider lack of novelty as a criterion for rejection and its main objective is to overcome this publication bias stressing on publishing good research. We think that our study lies within this domain. Furthermore, methods of studies are usually used before and not novel.
4. The reviewers asked for more details on the way the questionnaire was developed which we have now added to the methods section as requested (Page 6, lines 120-134).
5. The reviewer has the opinion that research methods should be the same in normal and disaster situations which we completely disagree with. The senior author of the current paper (F A-Z) is an international expert with extensive practical experience in disaster medicine. This point has now been more clarified and referenced in the limitation section (Page 22, lines 324- 328 and Page 23, lines 329-331).

Finally, it will be assuring for the reviewer that his/her anonymous comments will be published alongside the publication of this paper beside our response so these comments and concerns will be shared with the general readers.

**Reviewers' comments**

**Reviewer 1**

**Comment:** All corrections have been done well. All parts like introduction, methodology, findings and discussion include details information.

**Answer:** Thank you for your previous advice and highly encouraging comment. Nothing to answer.

**Reviewer 2**

**Comment 1:** **a)** Some comments have not been addressed suitably including the discussion, methods, …. And the revision could not convince me for further steps. The rationale of conducting this research was not stated clearly. What was the main necessities of doing this study? There were many similar studies in this field and the results may be predictable. **b)** The previous study (ref. number 28) is very similar to this study so what was the need?

6. **Answers: a)** We completely disagree with this comment. The introduction has a classical reversed pyramid approach in which we started broad and then narrowed the discussion gradually to reach a well-defined aim at the end of the introduction. **b)** Our study is different from reference 28 which studied a single university in UAE with both medical and non-medical students while we have studied medical and allied health students in all UAE. Our study has much generalizability compared with reference 28. We have clarified this in the introduction (Page 4, lines 91-93).

**Comment 2:** **a)** The process of questionnaire’s modification was not clearly discussed. **b)** Additionally, the methods section is too common and repeated and there is not enough novelty in the research methodologically.

7. **Answer:** Thank you for your concerns**.**
a) The details of how the questionnaire was developed has now been added to the methods section as requested (Page 6, lines 120-134).
b) We are aware of this point. We have submitted our paper to PLOS ONE because it does not consider lack of novelty as a criterion for rejection and its main objective is to overcome this publication bias stressing on publishing good research. We think that our study lies within this domain. Furthermore, methods of studies are usually used before and not novel.

**Comment 3:** Also, in my opinion, the last limitation may not be acceptable.

**Answer:** Thank you for your expressed opinion which is well appreciated. Kindly permit us to disagree with you. The senior author of the current paper (F A-Z) is an international expert with extensive practical experience in disaster medicine. This point has now been more clarified and referenced in the limitation section (Page 22, lines 324- 328 and Page 23, lines 329-331).

Thank you for your consideration for this revised manuscript. We hope that these changes will be adequate and answer the raised concerns, and that the manuscript finally finds a place in your reputable journal.

Yours Sincerely

Mohamud Sheek-Hussein and Fikri Abu-Zidan
